# Breast abscess due to *Listeria* sp: report of a rare case

**DOI:** 10.1186/1471-2334-14-S3-P20

**Published:** 2014-05-27

**Authors:** KV Ramana

**Affiliations:** 1 Department of Microbiology, Prathima Institute of Medical Sciences, Karimnagar, Andhrapradesh, India

## Background

*Listeria* sp. are facultative intracellular bacterial pathogens, which pose a potential public health problem related to consumption of contaminated food. Human listeriosis is clinically classified as perinatal listeriosis, neonatal listeriosis and adult listeriosis. Human infections caused by *Listeria sp.* present typically as meningitis. Other infections attributed to *Listeria sp.* include endocarditis, myocarditis, arteritis, pneumonia, pleuritis, cholecystitis, peritonitis, arthritis, osteomyelitis, sinusitis, otitis, conjunctivitis and ophthalmitis. We report a case of breast abscess in a young lactating patient.

## Case presentation

A lactating 21 year-old lady presented to hospital with complaints of a lump in her right breast. On observation the size of the lump was 3cmX1cm. Gram stain of the aspirated pus revealed short gram positive bacilli, and culture on blood agar grew small, round translucent beta hemolytic colonies with no growth on MacConkey agar. Conventional biochemical reactions showed that the isolated bacteria was motile at 25°C and non-motile at 37° C and cold enrichment at 40 °C, 10% salt (NaCl) tolerance test and CAMP test were positive. Histopathological study of the tissue biopsy revealed extensive inflammation with no signs of granuloma. Empirical therapy including Amoxy clav 1.2 mg TID and 400 mg Ciprofloxacin TID was started. The patient responded well and had an uneventful recovery.

## Conclusion

Human infections with *Listeria* sp. remain under reported owing to its unusual clinical presentation and complex physiological and biochemical characters. Clinical microbiologists should be proactive in the laboratory identification of *Listeria* sp. as most isolates of Listeria are ignored as laboratory contaminants.

